# CBX7 suppresses cell proliferation, migration, and invasion through the inhibition of PTEN/Akt signaling in pancreatic cancer

**DOI:** 10.18632/oncotarget.14037

**Published:** 2016-12-20

**Authors:** Sujie Ni, Hongwei Wang, Xiaolin Zhu, Chunhua Wan, Junfei Xu, Chen Lu, Li Xiao, Jiaqi He, Chongyi Jiang, Wei Wang, Zhixian He

**Affiliations:** ^1^ Department of Medical Oncology, Affiliated Hospital of Nantong University, Nantong University, Nantong 226001, China; ^2^ Bilary and Pancreatic Center, Huadong Hospital of Fudan University, Fudan University, Shanghai 200040, China; ^3^ Department of Gastroenterology, Affiliated Hospital of Nantong University, Nantong University, Nantong 226001, China; ^4^ Department of Nutrition and Food Hygiene, School of Public Health, Nantong University, Nantong 226001, China; ^5^ Department of General Surgery, Affiliated Hospital of Nantong University, Nantong University, Nantong 226001, China; ^6^ Department of Pathology, Huadong Hospital of Fudan University, Fudan University, Shanghai 200040, China

**Keywords:** CBX7, pancreatic cancer, prognosis, tumor suppression, PTEN

## Abstract

Chromobox protein homolog 7 (CBX7), one of the polycomb group (PcG) proteins, is a transcriptional repressor involved in the regulation of cell proliferation and senescence. In the present study, we showed that CBX7 negatively regulates the proliferation, viability, chemoresistance, and migration of pancreatic cancer cells. Overexpression of CBX7 significantly inhibited the proliferation of pancreatic cancer cells *in vitro* and *in vivo*. Depletion of CBX7 facilitated their growth. CBX7 also impaired the viability and chemoresistance of pancreatic cancer cells. Transwell assays showed that CBX7 reduces the migratory capacity of pancreatic cancer cells. Of note, CBX7 reduced PTEN/Akt signaling in pancreatic cancer cells by increasing PTEN transcription, suggesting involvement of PTEN/Akt pathway in the tumor suppressive activity of CBX7. In addition, immunohistochemical analysis the CBX7 and PTEN expression in 74 surgically resected pancreatic ductal adenocarcinoma (PDAC) specimens revealed that CBX7 expression is significantly downregulated in pancreatic ductal adenocarcinoma, compared to normal pancreatic tissues. Reduced expression of CBX7 and PTEN was associated with increased malignancy grade in pancreatic adenocarcinoma, whereas maintenance of CBX7 and PTEN expression showed a trend toward a longer survival. These findings suggest CBX7 is an important tumor suppressor that negatively modulates PTEN/Akt signaling during pancreatic tumorigenesis.

## INTRODUCTION

Approximately 90% of all pancreatic cancer cases are fatal, making it one of the most lethal malignancies and a major worldwide health burden [1, 2]. Despite 50 years of active research, the prognosis for pancreatic cancer remains extremely poor with few therapeutic options [3]. It is estimated that pancreatic cancer has a median survival of 6 months and a 5-year survival rate of less than 5% [4]. Surgical resection at an early stage remains the only chance to cure pancreatic cancer, but the vast majority of cases are diagnosed at an advanced unresectable stage, past the window for radical surgery [5]. Therefore, there is an urgency to find additional novel and effective agents to manage this dreadful disease.

Polycomb group (PcG) proteins are a class of epigenetic regulators, and typically form multiprotein complexes to exert their functions in regulating cell proliferation, senescence and tumorigenesis via well-known growth regulatory pathways [6]. Biochemical and genetic studies indicate that PcG proteins act as part of at least two high molecular weight complexes, Polycomb repressive complexes 1 and 2 (PRC1 and PRC2). The components of the PRC1 complex are the mammalian homologs of Drosophila Polycomb (Pc), Posterior sex combs (Pscs), Sex combs extra (Sce), and Polyhomeiotic (Ph) [7]. CBX7 is a Pc homolog consisting of a conserved chromodomain near the N-terminus and a Polycomb box domain in the C-terminal region [8]. CBX7 inhibits cellular senescence and extend the lifespan of normal human cells by downregulating the expression of INK4a/ARF, and cooperate with c-Myc in lymphomagenesis [7, 9, 10]. However, other studies have shown that CBX7 expression is inversely correlated with malignancy grade and neoplasia stage in thyroid neoplasia [11]. Likewise, a positive correlation between loss of CBX7 expression and poor prognosis was found in human colon and breast carcinomas [12, 13]. These results indicate that the precise role of CBX7 in cancer development may vary between different cancer types. Despite the fact that loss of CBX7 expression has been associated with enhanced malignancy in pancreatic cancer, the underlying molecular mechanism remains to be elucidated [14]. In this study, we investigate the effect of CBX7 on pancreatic cancer development and explore the underlying molecular mechanisms.

## RESULTS

### CBX7 inhibits the growth and colony formation of pancreatic cancer cells

**Figure 1:**
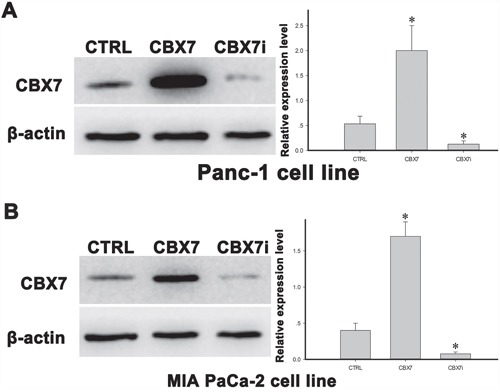
The establishment of stable CBX7-overexpressing and CBX7-knockdown pancreatic cell lines Panc-1 and MIA PaCa-2 pancreatic cancer cells were infected with Control (CTRL), CBX7-overexpressing (CBX7) and CBX7-interfering (CBX7i) lentivirus. The cells were later subjected to puromycin selection to obtain stably infected cell lines. Western blot analysis was conducted to determine the protein level of CBX7 in Panc-1 **A.** and MIA PaCa-2 **B.** cells. (* P<0.05, compared with the CTRL group).

To elucidate the potential role of CBX7 in pancreatic cancer development, we employed a lentivirus-based expression system to overexpress or deplete CBX7 in pancreatic cancer cells. Control (Con), CBX7-expressing, and CBX7-siRNA lentiviruses were packaged and used to infect Panc-1 and MIA PaCa-2 pancreatic cancer cells. The cells were subjected to puromycin selection to obtain CBX7 stably expressing or depleted cell lines. Western blotting results revealed that the cellular level of CBX7 was markedly upregulated following CBX7-lentivirus infection in both Panc-1 and MIA PaCa-2 pancreatic cancer cells (Figure 1A and 1B). Likewise, infection with CBX7-siRNA lentivirus significantly reduced CBX7 in Panc-1 and MIA PaCa-2 cells, compared with cells infected with control virus (Figure 1A and 1B).

**Figure 2:**
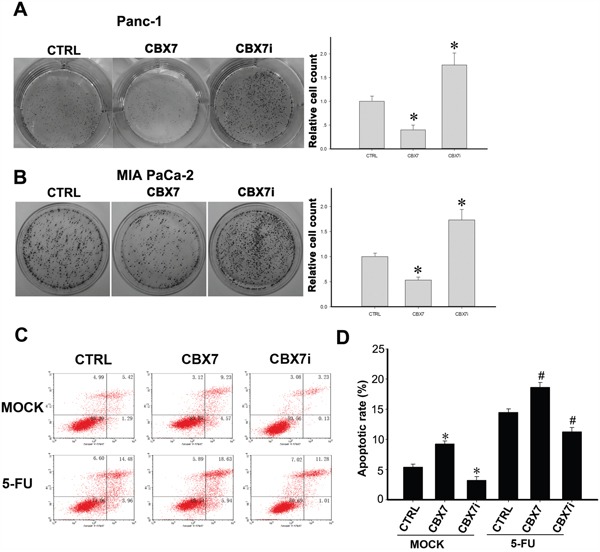
Determination of the colony formation capacity, viability, and chemoresistance of CTRL, CBX7, and CBX7i pancreatic cancer cells 500 cells of the indicated groups of Panc-1 **A.** and MIA PaCa-2 **B.** cells were seeded into 6-well plates and incubated for 10 days. The colonies formed were fixed by paraformaldehyde and stained with 0.5% crystal violet. C. Annexin-V/PI analysis of cell viability and chemoresistance of CTRL, CBX7, and CBX7i pancreatic cancer cells. Pancreatic cancer cells were treated with or without 50 μg/ml 5-FU and subjected to annexin-V/PI apoptotic assay. D. The bar chart showed the apoptotic rate of the indicated groups. (* P<0.05, compared with the control groups (CTRL); # P<0.05, compared with 5-FU-treated CTRL cells).

Because CBX7 is reportedly involved in pancreatic cancer malignancy, we analyzed whether the colony formation capacity was altered in these cells. As shown in Figure 2A and 2B, CBX7 overexpression markedly decreased the number of colonies both in Panc-1 and MIA PaCa-2 cells. Coincidently, depletion of CBX7 increased the number of colonies, compared with the control group.

### CBX7 impairs the viability and chemoresistance of pancreatic cancer cells

The impact of CBX7 on the viability and chemoresistance of PDAC cells was also assessed. We found that overexpression of CBX7 reduced the viability of Panc-1 cells, whereas depletion of CBX7 led to increased viability of PDAC cells (Figure 2C and 2D). Similarly, we found that CBX7 overexpression promoted 5-Fluorouracil (5-FU)-induced apoptosis of Panc-1 cells, whereas CBX7-depleted cells showed decreased apoptosis following 5-FU exposure (Figure 2C and 2D).

### CBX7 inhibits the migration of pancreatic cancer cells

**Figure 3:**
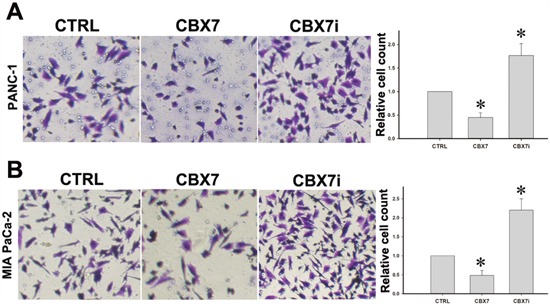
CBX7 attenuates the migration of pancreatic cancer cells Transwell migration assay was performed to determine the influence of CBX7 on the migration of pancreatic cancer cells. Panc-1 **A.** and MIA PaCa-2 **B.** PDAC cells were subjected to migration assay using Corning chamber to test the migration capacities of CTRL, CBX7, and CBX7i cells. (* P<0.05, compared with the CTRL group).

Invasion and metastasis are the most important hallmarks of cancer cells. We used transwell chamber assay to explore the impact of CBX7 on the migratory capacity of pancreatic cancer cells. As shown in Figure 3A and 3B, the migratory phenotype of Panc-1 and MIA PaCa-2 was markedly impaired by CBX7 overexpression. These findings suggested that CBX7 inhibited the migratory capacity of pancreatic cells *in vitro*.

### CBX7 suppresses the growth and proliferation of Panc-1 *in vivo*

**Figure 4:**
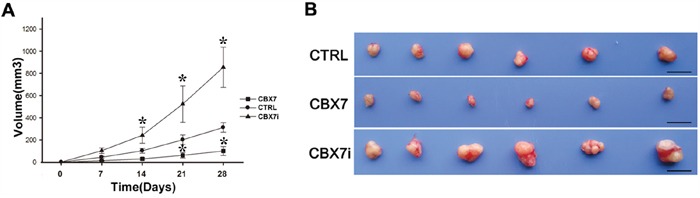
Analysis of the in vivo tumorigenesis of CTRL, CBX7, and CBX7i pancreatic cancer cells using xenograft tumor assay CTRL, CBX7, and CBX7i pancreatic cancer cells were subcutaneously injected into the flanks of nude mice. The tumor volumes were measured weekly. **A.** The growth curves of CTRL, CBX7, and CBX7i tumors were measured and presented. (* P<0.05, compared with the control groups (CTRL)). **B.** The representative images of xenograft tumors in the indicated groups (Scale bar, 10 mm).

Based on the above findings, we conducted xenograft tumor experiments to verify the effect of CBX7 on pancreatic cancer growth *in vivo*. Lenti-CBX7, Lenti-control, and Lenti-siCBX7 Panc-1 cells were injected subcutaneously into the right limb of nude mice. Four weeks later, CBX7-overexpressing xenograft tumors displayed statistically significant lower weights than the control group. In keeping with this data, the mice injected with Lenti-siCBX7 panc-1 cells had larger xenograft tumors than the control group (Figure 4).

### CBX7 inhibits PI3K/Akt signaling pathway in pancreatic cancer by activating PTEN transcription

**Figure 5:**
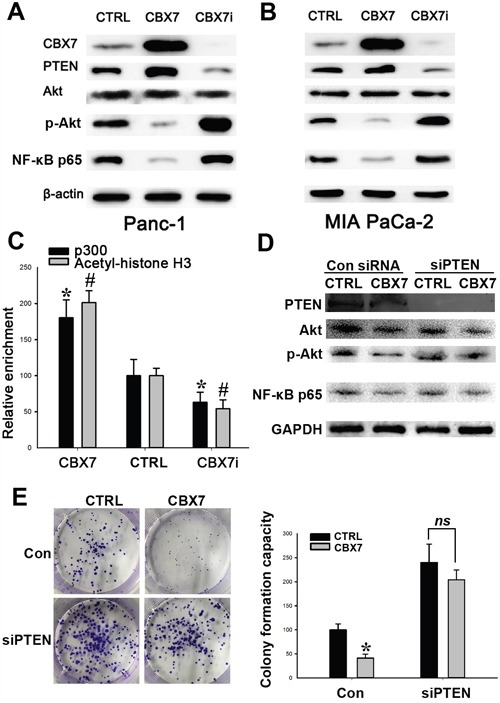
CBX7 suppresses PTEN/Akt/NF-κB signaling in pancreatic cancer cells CTRL, CBX7, and CBX7i Panc-1 **A.** and MIA PaCa-2 **B.** cells were subjected to western blot to determine the expression of PTEN, p-Akt, and NF-κB p65. **C.** CBX7 promotes p300-PTEN promoter binding and Histone H3 acetylation in pancreatic cancer cells. CTRL, CBX7, and CBX7i Panc-1 cells were subjected to CHIP assay to determine the enrichment of p300 and acetylated Histone H3 on PTEN promoter. (*,# P<0.05, compared with the control group.) **D.** Depletion of PTEN abrogated the suppressive effect of CBX7 on PTEN/Akt/NF-κB pathway. CTRL and CBX7 panc-1 cells were transfected with PTEN siRNA pool. 48 h after transfection, the cells were subjected to western blot analysis to determine the levels of PTEN, p-Akt and NF-κB p65 in the indicated groups of cells. **E.** Depletion of PTEN significantly impaired the effect of CBX7 on the colonly-formaing capacities of pancreatic cancer cells. Determination of the colony-forming capacities of the indicated groups of cells. (* P<0.05, compared with the control group; ns, no statistical difference).

To explore the potential mechanisms underlying the tumor-suppressive role of CBX7 in pancreatic carcinogenesis, we further analyzed the signaling pathways that might be affected by CBX7 in pancreatic cancer cells. Microarray and GO-pathway analysis predicted that CBX7 promotes the activation of PTEN and suppresses downstream PI3K/Akt pathway (Data not shown). Western blot analysis was performed to verify whether PTEN/Akt pathway was altered in response to CBX7 overexpression and depletion. We found that PTEN was upregulated in CBX7-overexpressing pancreatic cancer cells, compared with the control groups, whereas CBX7-depleted Panc-1 and MIA PaCa-2 cells exhibited reduced cellular PTEN (Figure 5A and 5B). Because PTEN mainly exerts its function through inhibiting downstream signal transducers, including Akt, NF-κB, and mTOR, we next examined the activities of downstream Akt and NF-κB pathways. Following CBX7 overexpression, the levels of cellular p-Akt and NF-κB p65 were significantly reduced in Panc-1 and MIA PaCa-2 cells, while CBX7 knockdown led to elevated levels of p-Akt and NF-κB p65 (Figure 5A and 5B). A recent study showed that CBX7 recruited p300 acetyltransferase to the DKK1 promoter region, driving DKK1 expression in breast cancer cells and leading to the impairment of Wnt/β-catenin signaling and breast tumorigenesis [15]. We speculate that CBX7 may work in a similar fashion to trigger the transcription of PTEN. Indeed, we confirmed that the enrichment of p300 on PTEN promoter was significantly enhanced in CBX7-overexpressing pancreatic cells (Figure 5C). Moreover, when PTEN was depleted, the impact of CBX7 on Akt/NF-κB pathway was significantly attenuated (Figure 5D). Accordingly, depletion of PTEN significantly diminished the effect of CBX7 on the colony formation capacity (Figure 5E). These findings suggest an involvement of p300 and histone H3 acetylation in CBX7-initiated PTEN expression.

### The correlation between CBX7 and PTEN expression in pancreatic cancer tissues

**Figure 6:**
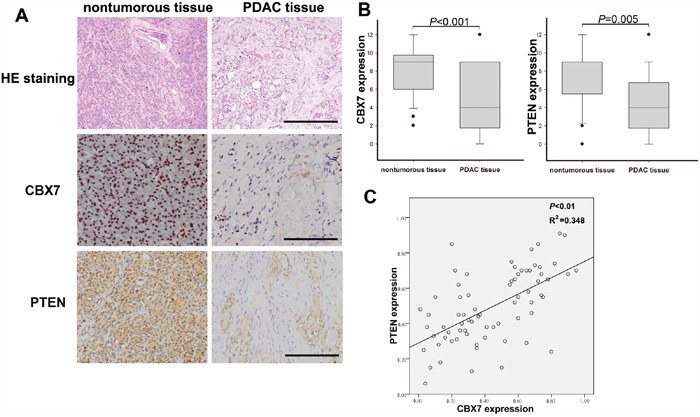
The expression pattern of CBX7 and PTEN in pancreatic cancer and adjacent non-tumor tissues and their correlation **A.** Hematoxylin-eosin (HE) staining and Immunohistochemical analysis of CBX7 and PTEN expression in human PDAC and non-tumor specimens (Scale bar, 100 μm). **B.** Statistical analysis of immunohistochemical scores of CBX7 and PTEN between pancreatic cancer and adjacent non-tumorous samples. (* P<0.05, compared with adjacent non-tumor tissues.) **C.** Linear regression analysis indicated a positive correlation between CBX7 and PTEN expression in pancreatic cancer tissues.

To further explore the involvement of CBX7 in pancreatic cancer development, we compared the expression of CBX7 and PTEN between PDAC and adjacent non-tumor tissues. 74 resected pancreatic cancer specimens were subjected to immunohistochemical analysis to determine the expression of CBX7 and PTEN. We found that CBX7 was abundantly expressed in most of the examined non-tumorous pancreatic tissues, while a small proportion of non-tumor tissues exhibited both cytoplasmic and nuclear expression of CBX7 (Figure 6A). In contrast, CBX7 was weakly expressed in most pancreatic cancer specimens. In some cases, a small proportion of PDAC cells showed modest nuclear expression of CBX7. PTEN was abundantly expressed in the cytoplasm of non-tumor pancreatic tissues and significantly downregulated in pancreatic cancer tissues (Figure 6A). Immunohistochemical evaluation indicated that CBX7 was significantly downregulated in PDAC tissues, compared with adjacent non-tumor tissues (Figure 6B). Next, we evaluated the immunohistochemical scores of CBX7 and PTEN expression by multiplying expression score and expression percentage in each PDAC tissue. The correlation between CBX7 and PTEN expression in PDAC tissues was analyzed using linear regression. In this way, we revealed a remarkable positive correlation between CBX7 and PTEN in these tissues, with a correlation coefficient of 0.348 (Figure 6C). These findings together suggested that the downregulation of CBX7 heavily contributes to the inactivation of PTEN during pancreatic cancer development.

### Prognostic significance of CBX7 expression in pancreatic cancer patients

**Figure 7:**
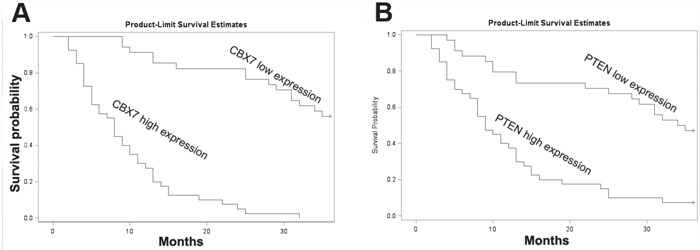
CBX7 and PTEN expression was strongly associated with the overall survival (OS) of 74 PDAC patients (P<0.05, log-rank test) **A, B.** Kaplan-Meier survival curve was presented according to CBX7 expression (A) and PTEN expression (B) assessed by IHC scores.

Next, we analyzed the correlation between CBX7 expression and the clinicopathological features of the patients. Patients were divided into low CBX7-expressing and high CBX7-expressing groups, according to the expression scores. As shown in Table 1, CBX7 expression was correlated with tumor grade, metastasis, and TMN stage, but not with other clinicopathological factors, including patients' age, gender, pT, and pN. Next, we determined the prognostic merit of CBX7 expression in the patients with pancreatic cancer. To this end, we plotted the survival curve of the patients using their follow-up data. PDAC patients with low expression of CBX7 showed a trend towards significantly worsened survival time, compared with those with high CBX7 expression (Figure 7). Similarly, we found that patients with low PTEN expression showed a similar dismal prognosis, compared those with high PTEN expression. Moreover, multivariate Cox regression analysis revealed that CBX7 could serve as independent prognostic indicator for predicting the survival of patients (Table 2).

**Table 1:** The correlation between CBX7 expression and clinicopathological factors in 74 pancreatic cancer patients

Variable	CBX7 weak expression		CBX7 strong expression		χ^2^	P value
	Percent		Percent			
**Age(years)**						
<60	11	27.5	13	38.2	0.967	0.995
≥60	29	72.5	21	61.8		
**Gender**						
Male	19	47.5	25	73.5	5.166	0.330
Female	21	52.5	9	26.5		
**Grade**						
Low differentiation	17	42.5	1	2.9	15.624	0.000*
M-H differentiation	23	57.5	33	97.1		
**pT**						
T1~T2	11	27.5	13	38.2	0.967	0.455
T3~T4	29	72.5	21	61.8		
**pN**						
N_0_	27	67.5	23	67.6	0.000	1.000
N_1_	13	32.5	11	32.4		
**Metastasis**						
Negative	32	80.0	33	97.1	5.006	0.033*
Positive	8	20.0	1	2.9		
**TNM stage**						
Ia~IIb	32	80.0	33	97.1	5.006	0.033*
III~IV	8	20.0	1	2.9		

**Table 2:** Multiple Cox regression analysis of CBX7 expression as an independent prognostic factor on 74 pancreatic cancer specimens

Characteristic	Univariate Cox regression			Multivariate Cox regression		
	HR	95%CI	*p* value	HR	95%CI	*p* value
Gender	1.058	0.574-1.950	0.856			
Age	1.171	0.597-2.297	0.646			
Location	0.955	0.503-1.811	0.887			
Size	0.318	0.087-1.159	0.082			
Histological differentiation	2.506	1.223-5.135	0.012			
TNM stage	5.949	1.543-22.933	0.0096	1.633	0.537-4.965	0.388
CBX7	0.098	0.043-0.222	<0.0001	0.094	0.046-0.190	<0.0001

Cox proportional hazards regression analysis revealed that CBX7 could serve as an independent prognostic factor of pancreatic cancer.

These reverse associations remained after adjusting for age (< 60 years old and ≥60 years old), gender, TNM, differentiated, tumor size (>2 cm and < 2cm), as well as tumor location. Comparing to patients with weakly positive status, the hazard ratios is 0.098 (0.043-0.222) for CBX7, p < 0.0001.

## DISCUSSION

Due to late diagnosis and limited therapeutic strategies, the prognosis of pancreatic cancer remains dismal and largely unchanged for decades. Accumulating evidence indicates that targeted therapy may be promising significance for the treatment of pancreatic cancer. Therefore, it is crucial to discover novel molecular targets in pancreatic cancer and evaluate their therapeutic merits [11, 16]. In the present study, we found that the GpC protein CBX7 was a tumor suppressor during pancreatic tumorigenesis. Increased expression of CBX7 was inversely correlated with tumor grade and metastasis in pancreatic cancer. In addition, we identified that CBX7 inhibited the proliferation, migration, and invasion of cultured pancreatic cancer cells. Finally, our data showed that CBX7 overexpression suppressed pancreatic cancer progression *in vivo*. These findings demonstrate that CBX7 is a negative regulator of pancreatic cancer development, potentially guiding the development of molecularly targeted therapy for this deadly disease.

Multiple studies have revealed that CBX7 may have diverse roles in different cancer types. In addition, CBX7 may initiate T-cell lymphomagenesis and cooperate with c-Myc to produce highly aggressive B-cell lymphomas [7]. Moreover, it has also been shown that CBX7 expression facilitates the survival of the mouse embryonic fibroblasts [17]. These results suggest that CBX7 contributes to the development of carcinogenesis in various tissues. However, other publications propose CBX7 as a potential tumor suppressor. It was observed that loss of CBX7 expression correlated with aggressive phenotypes in thyroid carcinoma and colorectal carcinoma [11, 13]. Therefore, we speculate that the function of CBX7 varies among different tumors, depending on the cellular events and signaling pathways that CBX7 initiates [18]. In the present study, we provided *in vitro* and *in vivo* evidence to support a tumor-suppressive role of CBX7 in pancreatic cancer. In this regard, we found that CBX7 expression is decreased in pancreatic cancer cell lines and pancreatic tissues. Restoration of CBX7 expression impairs the tumor growth, migration, and invasion of pancreatic cancer *in vivo* and *in vitro*, whereas stable knockdown of CBX7 expression results in opposite phenotypes. Of great importance, we found expression of CBX7 was significantly reduced in PDAC tissues relative to normal pancreatic tissues. CBX7 protein expression appeared to be inversely correlated with the malignancy grade of pancreatic cancer. Loss of CBX7 expression also showed a trend towards worsened prognosis, and most of the patients with poor survival outcome exhibited negative expression of CBX7.

Serving as a transcriptional regulator, CBX7 has been implicated in the transcriptional regulation of various oncogenes and tumor suppressors. CBX7 promotes the proliferation of normal and tumor-derived prostate cells by repressing the transcription of the tumor suppressors p16^Ink4a^ and p14^ARF^ [19]. Forzati F et al. reported that CBX7 suppressed the expression of cyclin E by forming a transcriptional-suppressing complex with histone deacetylase HDAC2 on the cyclin E promoter [20]. In addition to transcriptional suppression, CBX7 may initiate the transcription of target genes through the recruitment of transcriptional coactivators. Our studies showed that CBX7 could facilitate the transcription of PTEN by promoting p300-PTEN promoter interaction and subsequent histone acetylation. PTEN is a classical tumor suppressor and plays a pivotal role in the suppression of various cancer types, including pancreatic cancer. Early studies showed that aberrant PTEN expression leads to the activation of PI3K/Akt signaling pathway, targeting NF-κB and c-Myc transcription factors [21]. Later investigations indicated that PTEN loss-of-function promotes an NF-κB-Cytokine network and tumor-favorable microenvironment [22]. Studies also found that PTEN might regulate angiogenesis, chemoresistance, and tumor stemness in human pancreatic cancer cells [22–24]. Loss of PTEN accelerates pancreatic tumorigenesis in Kras-mutated mice [25]. These findings highlight the importance of PTEN loss-of-function in pancreatic cancer development. Our studies showed that CBX7 could regulate PTEN transcription in pancreatic cancer cells. Depletion of PTEN attenuated the influence of CBX7 on colony formation capacity in pancreatic cancer cells. In addition, using linear regression analysis, we revealed that the expression of CBX7 was positively correlated with PTEN in patients with pancreatic cancer. These findings implicated a key involvement of PTEN in CBX7-mediated tumor suppression.

It has been well-documented that PTEN/Akt signaling is a master intracellular pathway in cancer biology. PTEN/Akt signaling affects a wide range of cancer cell behavior, including cell viability, senescence, proliferation, migration, and invasion by regulating the activities of various transcription factors and signaling molecules, including NF-κB, β-catenin, FOXOs, and mTOR [26]. NF-κB is a classical oncogenic pathway that is tightly regulated by the PTEN/PI3K/Akt pathway. Akt directly phosphorylates IκB to trigger rapid degradation of IκB, which eventually leads to the phosphorylation and nuclear accumulation of NF-κB proteins [27, 28]. NF-κB drives cell proliferation, chemoresistance and migration through the transcription of various target genes, including Cyclin D1, c-myc, Bcl-2, and Matrix Metalloproteinases (MMPs) [29, 30]. In our study, we found that restoration of CBX7 resulted in NF-κB expression, which suggested that the regulation of PTEN/Akt and downstream NF-κB pathways might be one of the important mechanisms underlying CBX7's tumor suppressive role in pancreatic adenocarcinoma.

In conclusion, we found that CBX7 plays a tumor-suppressive role in pancreatic cancer by the regulation of the PI3K/Akt signaling pathway. Loss of CBX7 expression is associated with increasing malignancy grade in pancreatic adenocarcinoma, whereas the maintenance of CBX7 expression correlated with longer survival.

## MATERIALS AND METHODS

### Cell lines and transfection

Human pancreatic cancer cell lines Panc-1 and MIA PaCa-2 were purchased from the Cell Bank of the Chinese Academy of Sciences (Shanghai, China). Human normal pancreatic cell line HPDE6-C7 was obtained from the Pancreatic Cancer Institute, Fudan University. These cell lines were cultured in DMEM supplemented with 10% fetal bovine serum (FBS) and antibiotics.

Transfection of PTEN siRNA oligos pool. The three target sequences of the PTEN siRNA were 5′-GUA UAG AGC GUG CAG AUA A-3′ (siRNA#1) and 5′-AGA GUU GCA CAA UAU CCU U-3′ (siRNA#2) and 5′-GUC AGA GGC GCU AUG UGU A-3′ (siRNA#3) which were designed and synthesized by Biomics (Shanghai, China). The control nucleotide sequence of siRNA was 5′-UUC UCC GAA CUUGUC ACG U-3′. Panc-1 and MIA PaCa-2 cells were plated onto a 6-well or 96-well plates (Corning, NY, USA) at 40–60 % confluence the day before transfection. Twenty-four hours later, the PTEN-targeting and control siRNA oligos were transfected into the cells using Lipotransfectamine 2000 (Invitrogen, Carlsbad, CA) in accordance with the manufacturer's instructions.

### The establishment of stable CBX7-overexpressing and -knockdown PDAC cells

CBX7-overexpressing lentivirus construct was obtained by amplifying the coding sequence of human CBX7 and cloning it into a Lv6/Puro vector (GenePharma, Shanghai, China). The lentivirus was packaged by co-transfecting Lv6-CBX7 and helper vectors (pGag/Pol, pRev, pVSV-G) into 293T cells. 48 h after transfection, CBX7-overexpressing lentivirus was collected, centrifugated at 800 g, 4 °C for 10 min and used for PDAC cell infection. The infected PDAC cells were selected with 1 μg/ml puromycin (Sigma, St. Louis, MO, USA) to obtain stable CBX7-overexpressing PDAC cells. The expression of CBX7 was verified using Western blot analysis.

To stably silence CBX7 expression in PDAC cells, three CBX7-specific siRNAs were designed and synthesized by GenePharm (Shanghai, China). The siRNA oligo (5'-GCT GGT TCT GGG AGT TAA AGG-3') with best interference efficiency was chosen for lentivirus packaging by GenePharma (Shanghai, China). PDAC cells were infected with CBX7-shRNA lentivirus and subjected to 1 μg/ml puromycin selection. The interference efficiency of CBX7-shRNA lentivirus was determined using western blot analysis.

### Colony formation assay

Panc-1 and MIA PaCa-2 cells were seeded into 35 mm wells at a density of 500 cells per well. 2 weeks after cell seeding, each well was stained with crystal violet and examined using light microscopy.

### Cell migration assay

Cell migration ability was analyzed by the transwell chamber assay. Cells were plated in serum-free medium, and medium containing 10% FBS in the lower chamber served as the chemoattractant. After 24 hours of incubation, the cells that did not migrate or invade through the pores were carefully wiped out with cotton wool. Then, the inserts were stained with 20% methanol and 0.2% crystal violet, imaged, and counted with an IX71 inverted microscope (Olympus, Tokyo, Japan).

### Protein extraction and western blotting

The whole cell lysates were harvested using RIPA lysis buffer supplemented with a protease inhibitor cocktail (Sigma, St. Louis, MO, USA), and Western blotting analyses were performed as described. Protein extraction of cultured cells was performed using a protein extraction kit from WELL BioTech (Shanghai, China) in accordance with the manufacturer's instruction. Antibodies against CBX7, PTEN, total-Akt, phospho-Akt (p-Akt) and NF-κB p65 were purchased from Abcam (Cambridge, MA), and used at 1: 1000 dilution. Anti-β-actin antibody (Santa Cruz Biotechnology, Santa Cruz, CA) was used at 1:5000 dilution.

### Cell apoptotic analysis

Cell apoptotic analysis was performed using an Annexin-V/PI assay kit (Roche Diagnostics, Basel, Switzerland). Briefly, Panc-1 cells were treated with or without 50 μg/ml 5-Fluorouracil (5-FU) for 24 h. After that, the cells were digested with 0.25% Trypsin and washed with PBS. Next, the cells were stained with annexin-V-FITC/PI in accordance with the manufacturer's instructions. Finally, the samples were measured using an Accuri C6 flow cytometer (BD Biosciences, San Jose, CA, USA).

### Chromatin immunoprecipitation (CHIP) assay

ChIP assay was performed using the EpiTect ChIP qPCR Assay Kit (Qiagen, Hilden, Germany) in accordance with the manufacturer's instruction manual. The bound PTEN promoter sequence was verified using RT-PCR (Real-Time polymerase chain reaction) assay with DNA fragments immunoprecipitated by anti-p300 antibodies (ab14984, Abcam, Cambridge, MA), anti-acetyl-histone H3 (06-599; Upstate Biotechnology, Lake Placid, NY, USA), or control rabbit IgG. The DNA fragments were amplified by quantitative RT-PCR using Direct SYBR Premix (Takara, Dalian, China) on the LightCycler 480 Real-Time PCR System (Roche Diagnostics). The RT-PCR primers are 5′-CGG GCG GTG ATG TGG C-3′ and 5′-GCC TCA CAG CGG CTC AAC TCT-3′.

### Clinical samples

74 paraffin-embedded primary site specimens of pancreatic cancer were obtained from the archives of Huadong Hospital, Fudan University for further immunohistochemical (IHC) analysis. The clinicopathologic variables were obtained from the medical records, and the disease stages of the patients were classified according to the 2014 NCCN Pancreatic Cancer TNM staging system.

### Immunohistochemistry and Immunohistochemical evaluation

The paraffin-embedded tissues were cut into 4 μm paraffin sections. Immunohistochemical (IHC) analysis was performed to determine the expression of CBX7 in pancreatic cancer primary lesions and adjacent non-tumorous tissues. IHC was performed using a highly sensitive streptavidin-biotin-peroxidase detection system. To quantify CBX7 protein expression, both the intensity and extent of CBX7 immunoreactivity were evaluated and scored. All sections were examined by two independent pathologists in a blinded fashion. IHC intensity was scored as follows: 0, negative staining; 1, weak staining; 2, moderate staining; 3, strong staining. The scores of the extent of immunoreactivity ranged from 0 to 3, according to the percentage of cells that exhibited positive staining in each microscopic field of view (0, <25%; 1, 25%-50%; 2, 50%-75%; 3, 75%-100%). A final score ranging from 0 to 9 was achieved by multiplying the scores for intensity and extent. CBX7 expression level was considered high when the final scores were ≥ 4 and low when the final scores were < 4.

### Xenograft tumor model

Panc-1 cells were injected subcutaneously into the flanks of nude mice (n=6 in each group). The tumors were measured weekly using a Vernier caliper and the volumes of tumors were calculated using the following formula: volume (mm^3^) = length × width^2^× 0.5. 4 weeks after cell injection, mice were sacrificed by cervical dislocation. The tumors were removed from the flanks of injected mice and examined using a stereoscopic microscope.

### Statistical analysis

All statistical analyses were performed using the SPSS 16.0 software package, and two-tailed P values of less than 0.05 were considered significant. Differences among variables were measured by χ2 analysis or Student's t tests. Quantitative data were expressed as the mean ± SD unless otherwise indicated. In the set of IHC assays of paraffin-embedded tissue samples, the Pearson χ2 test was used to determine the correlation between CBX7 expression and clinicopathologic characteristics.

### Study approval

All clinical samples were collected with the informed consent of the patients and study protocols that were in accordance with the ethical guidelines of the Declaration of Helsinki (1975) and were approved by the Institutional Medical Ethics Committee of Huadong Hospital, Fudan University. All experiments using animals were approved by the Animal Care and Use Committee of Fudan University.
